# BMP2-coprecipitated calcium phosphate granules enhance osteoinductivity of deproteinized bovine bone, and bone formation during critical-sized bone defect healing

**DOI:** 10.1038/srep41800

**Published:** 2017-01-31

**Authors:** Tie Liu, Yuanna Zheng, Gang Wu, Daniel Wismeijer, Janak L. Pathak, Yuelian Liu

**Affiliations:** 1 Department of Oral Implantology and Prosthetic Dentistry, Academic Centre for Dentistry Amsterdam (ACTA), Research Institute MOVE, University of Amsterdam and VU University Amsterdam, Gustav Mahlerlaan 3004, 1081 LA Amsterdam, the Netherlands; 2 Department of Oral Implantology, Hospital/School of Stomatology, Zhejiang University, Yan’an Road 395, 310006, Hangzhou, China; 3 School of Stomatology/Dental Clinic, Zhejiang Chinese Medical University, Binwen Road 548, Binjiang District, 310053, Hangzhou, China; 4 Department of Molecular and Cellular Pharmacology, School of Pharmaceutical Science and Technology, Tianjin University, 92 Weijin Road, 300072, Tianjin, China

## Abstract

Most materials used clinically for filling critical-sized bone defects (CSBD), such as deproteinized bovine bone (DBB), lack osteoinductivity so that their therapeutic effects are far from satisfactory. The effect of bone morphogenic protein 2 (BMP2)-coprecipitated biomimetic calcium phosphate granules (BMP2-cop.BioCaP) on osteoinduction of DBB graft(s) during CSBD healing is still unknown. We investigated whether BMP2-cop.BioCaP affects the osteoinductivity of DBB, bone formation, and foreign body reaction during CSBD healing. DBB + BMP2-cop.BioCaP, DBB, DBB + BMP2, DBB + BioCaP, and autologous bone grafts were implanted in the CSBD of sheep. Bone formation, DBB/BioCaP degradability, foreign body reaction, and osteoinductivity of DBB were analyzed histologically and histomorphometrically at week 4 and 8. Combination of BMP2-cop.BioCaP and DBB healed CSBD as effectively as autologous bone grafts. About 95% of the BMP2-cop.BioCaP had been degraded and replaced by new bone at week 8 in the DBB + BMP2-cop.BioCaP-group. Foreign body reaction was reduced in the DBB + BMP2-cop.BioCaP-group compared to the other groups. The independent use of the BMP2-cop.BioCaP did not achieve a satisfactory bone repair. In conclusion, the BMP2-cop.BioCaP showed good degradability and biocompatibility, and enhanced osteoinductivity of DBB during CSBD healing in sheep, suggesting BMP2-cop.BioCaP as a potential osteoinducer to enhance the therapeutic effects of the graft materials in clinic.

The essential part of treating bone fractures and defects is to achieve an adequate volume of bone tissue. When the bone defects are too large to heal by themselves, bone grafts are needed to fill the defect^1^,^2^. An autograft is still regarded as the gold standard in treatment since it provides an osteoconductive 3-dimensional scaffold for bone growth, osteogenic cells and osteoinductive growth factors^3^. However, autogenous bone is often associated with limitations such as the need for additional surgery, pain in the donor site, morbidity and a high and unpredictable resorption^4^,^5^. These limitations have led to a continual search for alternatives^6^,^7^. The development of numerous alternative materials for filling bone defects, such as allografts, xenografts, and synthetic materials have resulted from a better understanding of the biology of the healing of bones and technological development^8^,^9^. Most of these graft materials used clinically are osteoconductive, which enhances the migration of osteogenic cells. The capacity of the graft materials to induce osteogenesis is called osteoinductivity. However, these graft materials cause foreign body reaction and lack intrinsic osteoinductivity^8^,^10^. The foreign body reaction may significantly hinder the regeneration of bone and the osseointegration of material used for filling bone defects^10^. Therefore the therapeutic effects of alternative graft materials for filling bone defects during CSBD repair are far from satisfactory^8^.

DBB is widely used as a xenograft since its supply is unlimited, and it has the potential for reducing morbidity by eliminating additional surgery to obtain auto-graft^11^. DBB has excellent osteoconductive properties for serving as a scaffold for bone formation^12^ because it has a physico-chemical structure similar to that of natural bone^13^. Previous studies have demonstrated the efficiency of DBB in the repair of CSBD and in the augmentation of the maxillary sinus compared with other materials^14^,^15^. However DBB delays early bone formation in some cases which probably results from a lack of osteoinductivity^16^,^17^. On the other hand, both surgeons and patients would like to see the recovery phase shortened. It has been suggested that combining DBB with a particulate autograft would induce an adequate volume of bone tissue for an excellent restoration. This can provide osteogenic elements^12^,^18^, but the limitations related to autograft remain.

The application of osteogenic growth factors such as BMP2 by adsorbing them superficially onto DBB does not promote new bone formation in CSBD^19^,^20^ because it only gives a short-term burst of BMP2 release. Consequently, DBB has been considered as an unsuitable carrier for BMP2^21^. Therefore, a carrier coating that can release BMP2 sequentially might be a potential solution for sustained release. A calcium phosphate (CaP) coating with incorporated BMP2 enhances the osteoinductivity^22^. To further enhance osteoinductivity of the graft, we have recently developed a novel “osteoinducer” by biomimetically assembling CaP layer by layer and co-precipitating BMP2 into CaP granules (BMP2-cop.BioCaP). We found that a combination of the novel osteoinducer and DBB enhances ectopic bone formation in rat^23^. Similarly, a combination of BMP2-cop.BioCaP and biphasic calcium phosphate enhances cranial defect healing in the rat model^24^. However, the effect of combination of BMP2-cop.BioCaP and DBB on repair of CSBD in large animals had not been investigated.

We hypothesized that the BMP2-cop.BioCaP enhances the therapeutic effect of DBB during repair of CSBD. In order to prove this notion we tested the osteoinductivity, degradation, and foreign-body reaction of either BMP2-cop.BioCaP or its combination with DBB in CSBD repair in sheep.

## Results

### *In-vitro* characterization of BMP2-cop.BioCaP granules shows that incorporation of BMP2 does not alter the crystalline surface of BioCaP granules

**Figure 1:**
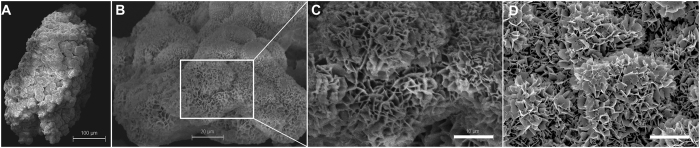
Incorporation of BMP2 did not affect the crystalline surface of BioCaP granules. (**A–C**) SEM micrograph of a BMP2-cop.BioCaP particle and surface. (**D**) SEM micrograph of BioCaP particle surface.

After three cycles of alternate immersion, the particle size increased from initial 5 to 20 μm to up to 250 to 1000 μm with a crystalline outer layer. According to our previous results^23^, 10 μg of BMP2 can be successfully incorporated into 0.07 cm^3^ of BioCaP, which induces efficient ectopic bone formation in rat. In the current study, ELISA results showed that each sample with BMP2-cop.BioCaP (0.07 cm^3^) contained 10.3 ± 1.9 μg BMP2 with an incorporation rate of 30.1 ± 5.7% (Supplementary Table 2). BMP2-cop.BioCaP granules were visualized by scanning electron microscopy (Fig. 1A,B), and high-resolution electron microscopic image showed that incorporation of BMP2 did not affect the crystalline surface of BioCaP granules (Fig. 1C,D).

### Combination of BMP2-cop.BioCaP and DBB enhanced bone formation

**Figure 2:**
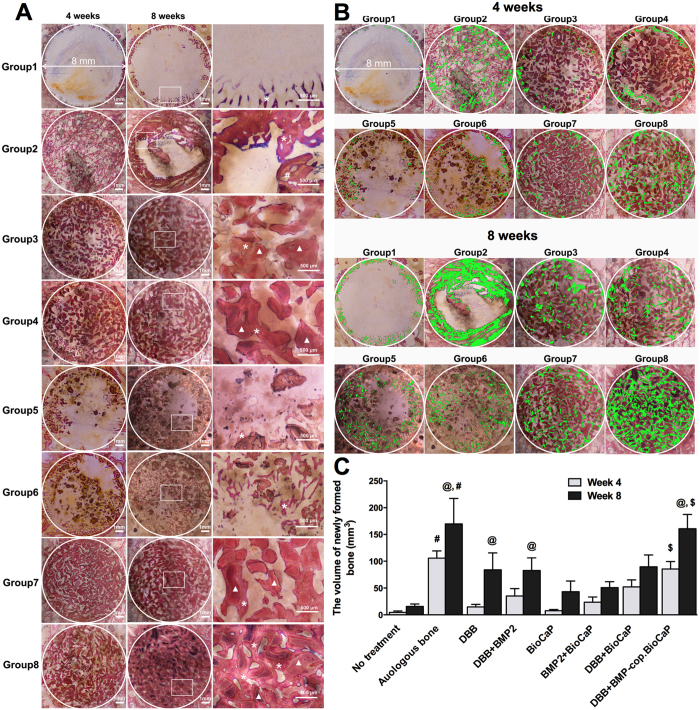
BMP2-cop.BioCaP enhanced bone formation in DBB grafted CSBD. (**A**) Representative histological images of the bone defect slices from 8 groups at week 4 and 8. (**B**) The newly formed bone in different groups at week 4 and 8 painted with pseudo color (green). (**C**) The volume of newly formed bone (mm^3^) during treatment of CSBD using different graft materials at week 4 and 8. Newly formed bone (*), DBB granules (white triangles). Significant difference between autologous bone-group and other groups, ^#^*p* < 0.05; between 4 and 8 weeks of same group, ^@^*p* < 0.05; DBB + BioC.BMP2 and other groups, ^$^*p* < 0.05.

Histological images show the bone-forming ability of different grafts (Fig. 2A). To visualize more clearly, we painted newly formed bone with pseudo color (green) (Fig. 2B). Higher volume of homogeneously formed new bone was observed in the DBB + BMP2-cop.BioCaP-group compare to other groups (Fig. 2A,B). Histomorphometry data showed that the volume of newly formed bone at week 8 was significantly higher in autologous bone-group (*p* < 0.0001), DBB-group (*p* < 0.0001), DBB + BMP2-group (*p* < 0.0013), and DBB + BMP2.cop-BioCaP-group (*p* < 0.0001) than at week 4 (Fig. 2C). At week 4, the volume of new bone in empty defect-group was 4.5 ± 2.7 mm^3^, whereas in the autologous bone-group it was 105.7 ± 13.6 mm^3^. The newly formed bone volume at week 4 in the DBB + BMP2-cop.BioCaP-group was 18.8-fold higher than in the empty defect-group, 5.8-fold higher than in the DBB-group, 2.4-fold higher than in the DBB + BMP2-group, 11-fold higher than in the BioCaP-group, 3.6-fold higher than in the BMP2-cop.BioCaP-group, and 1.6-fold higher than in the DBB + BioCaP-group, respectively (Fig. 2C). Similarly, the newly formed bone volume at week 8 in the DBB + BMP2-cop.BioCaP-group was 10.3-fold higher than in the empty defect-group, 1.9-fold higher than in the DBB-group, 1.9-fold higher than in the DBB + BMP2-group, 3.7-fold higher than in the BioCaP-group, 3.9-fold higher than in the BMP2-cop.BioCaP-group, and 1.8-fold higher than in the DBB + BioCaP-group (Fig. 2C). Interestingly, the newly formed bone volume in the DBB + BMP2-cop.BioCaP-group was similar to that in the autologous bone-group at week 4 and 8.

### BMP2-cop.BioCaP enhanced BioCaP degradation during CSBD repair

**Figure 3:**
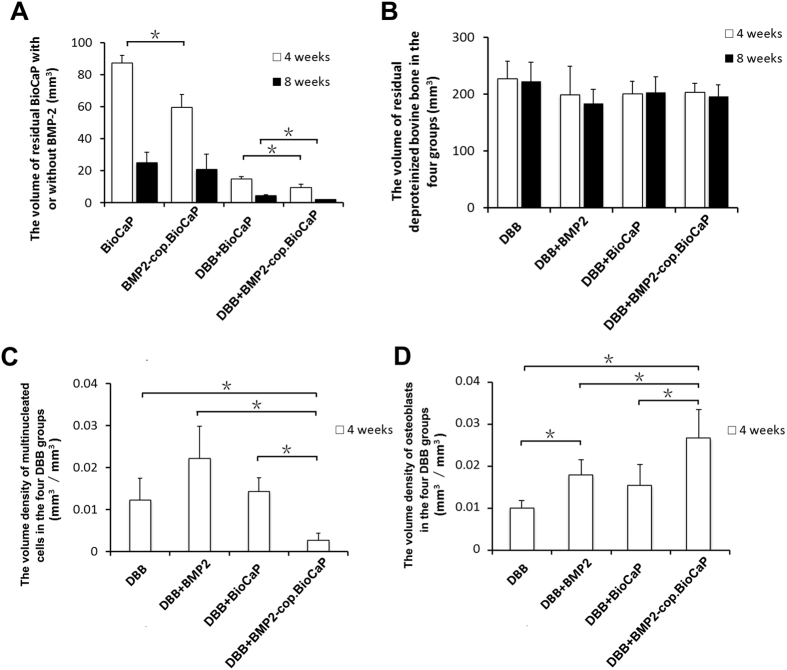
BMP2-cop.BioCaP enhanced degradation of BioCaP, and diminished DBB-induced foreign body reaction. (**A**) Residual volume of BioCaP at week 4 and 8. (**B**) Residual volume of BioCaP at week 4 and 8. (**C**) Foreign body reaction by DBB at week 4. (**D**) Osteoblast volume density at week 4. Values are mean ± SD from 6 bone defects. Significant effect of treatment, *p < 0.05.

We tested the effect of BMP2 and/or DBB on BioCaP degradation during CSBD healing. We found 31.6% less residual volume of BioCaP in BMP2-cop.BioCaP-group than in the BioCaP-group at week 4 (Fig. 3A). Similarly, the residual volume of BioCaP in the DBB + BMP2-cop.BioCaP-group was 37.5% less at week 4 and 55.6% less at week 8 than in the DBB + BioCaP-group (Fig. 3A). Combination of BMP2 and/or BioCaP with DBB did not affect the resorption of DBB at week 4 and 8 (Fig. 3B).

### BMP2-cop.BioCaP reduced foreign body reaction of DBB, and enhanced the volume density of osteoblasts at week 4

During tissue regeneration a higher number of multinucleated cells is an indicator of high inflammation. The volume density of multinucleated cells in the DBB + BMP2-cop.BioCaP-group was 78% less than in the DBB-group, 87.8% less than in the DBB + BMP2-group, and 81% less than in the DBB + BioCaP-group, respectively (Fig. 3C). The volume density of osteoblasts in the DBB + BMP2-group was 1.8-fold higher than in the DBB-group (Fig. 3D). Similarly, the volume density of osteoblasts in the DBB + BMP2-cop.BioCaP-group was 2.6-fold higher than in the DBB-group, 1.5-fold higher than in the DBB + BMP2-group, and 1.7-fold higher than in the DBB + BioCaP-group, respectively (Fig. 3D). Number of multinucleated cells or osteoblasts were too less to quantify the volume density at week 8.

### BMP2-cop.BioCaP enhanced osteoinductivity of DBB and facilitated CSBD healing

**Figure 4:**
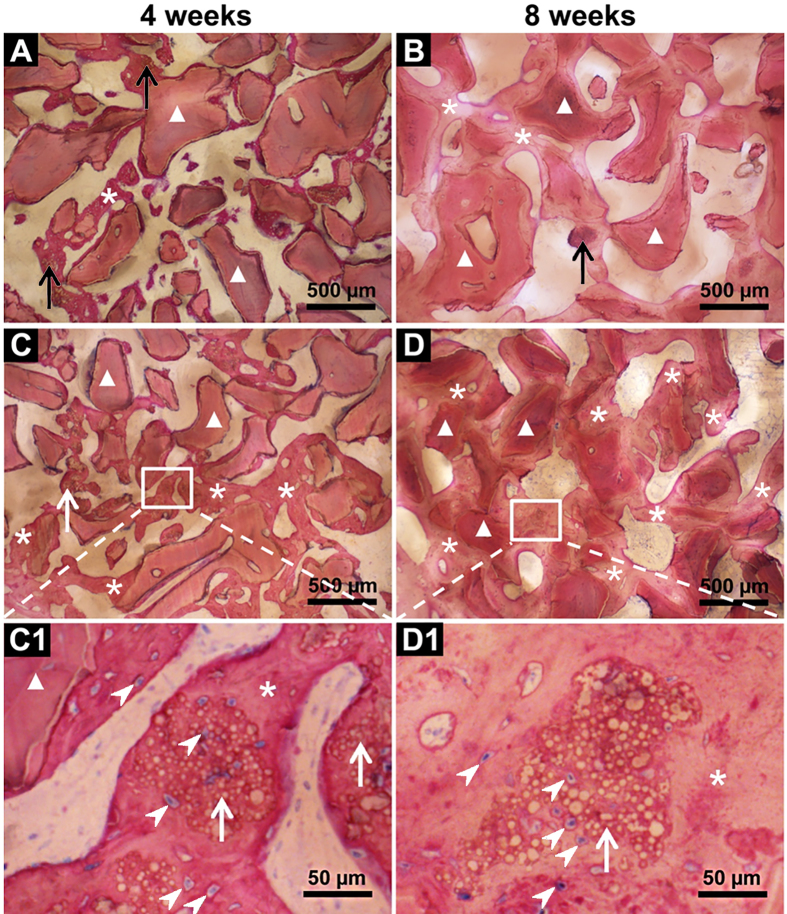
Representative histological images of the bone defect slices. (**A**) DBB + BioCaP-group at week 4. (**B**) DBB + BioCaP-group at week 8. (**C**) DBB + BMP2-cop.BioCaP-group at week 4. (**D**) DBB + BMP2-cop.BioCaP-group at week 8. (**C1**) high-resolution image of C. (**D1**) high-resolution image of D. BioCap (black arrow), BMP2-cop.BioCaP (white arrow), BioCaP or BMP2-cop.BioCaP in close contact with newly formed bone (*), osteocyte-like cells (white arrow heads), and DBB in close contact with newly formed bone (white triangle).

Histological examination showed unmineralized newly formed bone in the DBB + BMP2-cop.BioCaP-group after 4 weeks of implantation (Figs 2A,B and 4C,C1). The bone defect was fully filled with new bone and bone growth was observed throughout the space between DBB granules in DBB + BMP2-cop.BioCaP-group at week 8, indicating satisfactory healing of the critical size bone defects (Figs 2A,B and 4D,D1). In the DBB + BioCaP and DBB + BMP2-cop.BioCaP-groups, newly formed bone was always in close contact with the DBB surface and there was more mature bone at week 8 in comparison to week 4 (Fig. 4C1). Newly formed bone between DBB granules or deposited on DBB at week 4 had a woven appearance (Fig. 4A,C). Interconnected bone-DBB network was observed at week 8 (Fig. 4B,D). DBB granules were always encapsulated in bone. More bone growth was observed throughout the space between DBB granules in the DBB + BMP2-cop.BioCaP group (Fig. 4D,D1), compared with the DBB + BioCaP-group at week 4 and 8 (Fig. 4C,C1). Higher magnification images showed that BMP2-cop.BioCaP granules were observed constantly in close contact with new bone or completely encapsulated in the new bone at week 4 and 8 in the DBB + BMP2-cop.BioCaP-group (Fig. 4C1). Osteocyte-like cells were observed in close contact with the BMP2-cop.BioCaP particles at week 4 and 8, even in the interior of the particle between the small CaP spheres (Fig. 4C1). Newly formed bone in DBB + BioCaP and DBB + BMP2-cop.BioCaP-group is clearly visualized by green pseudo color (Supplementary Fig. 1).

**Figure 5:**
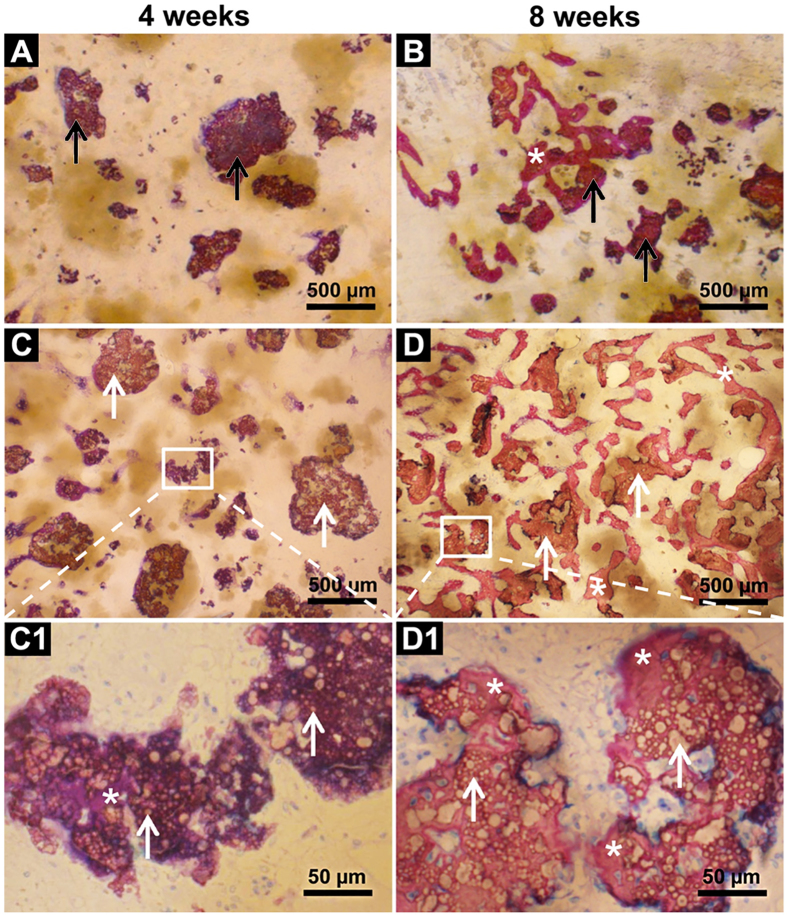
Representative histological images of the bone defect slices. (**A**) BioCaP-group at week 4. (**B**) BioCaP-group at week 8. (**C**) BMP2-cop.BioCaP-group at week 4. (**D**) BMP2-cop.BioCaP-group at week 8. (**C1**) High-resolution image of C. (**D1**) High-resolution image of D. BioCaP (black arrow), BMP2-cop.BioCaP (white arrow), BioCaP or BMP2-cop.BioCaP in close contact with newly formed bone (*), unmineralized newly formed bone (purple color), and mineralized and mature newly formed bone (reddish color).

In the BioCaP-group and the BMP2-cop.BioCaP-group, the residual BioCaP granules were not distributed uniformly in the bone defect. New bone formation was found mainly in the vicinity of BioCaP and in close contact with BioCaP granules at week 4 and 8 (Fig. 5A–D1). More newly formed bone was observed in the BMP2-cop.BioCaP-group than in the BioCaP-group at week 4 and 8. Higher-magnification images showed unmineralized newly formed bone with a deep purple color in between the BioCaP particles at week 4, and mature newly formed bone with a reddish color at week 8 (Fig. 5C1) of CSBD healing. Newly formed bone in DBB, DBB + BMP2, BioCaP, and BMP2 + Bio.CaP group is clearly visualized by green pseudo color (Supplementary Fig. 2).

### Observation of newly-formed bone, blood vessels, bone marrow, osteoblasts, and multinucleated cells during CSBD repair

**Figure 6:**
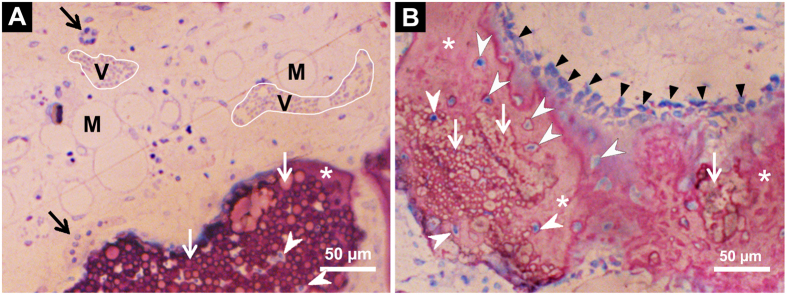
Representative histological sections showing BMP2-cop.BioCaP in the DBB + BMP2-cop.BioCaP-group at week 4. (**A**) The blood vessels in early stage of formation (V) and the bone marrow in early stage of formation (M) close to BMP2-cop.BioCaP. (**B**) An active phase of bone formation with osteoblasts (black triangles), osteocyte-like cells (white arrowheads) in close contact with or in the interior of the BMP2-cop.BioCaP particles. The slices were surface-stained with McNeal’s Tetrachrome, basic fuchsine and toluidine blue. BMP2-cop.BioCaP (white arrow), and multinucleated cells (black arrow).

**Figure 7:**
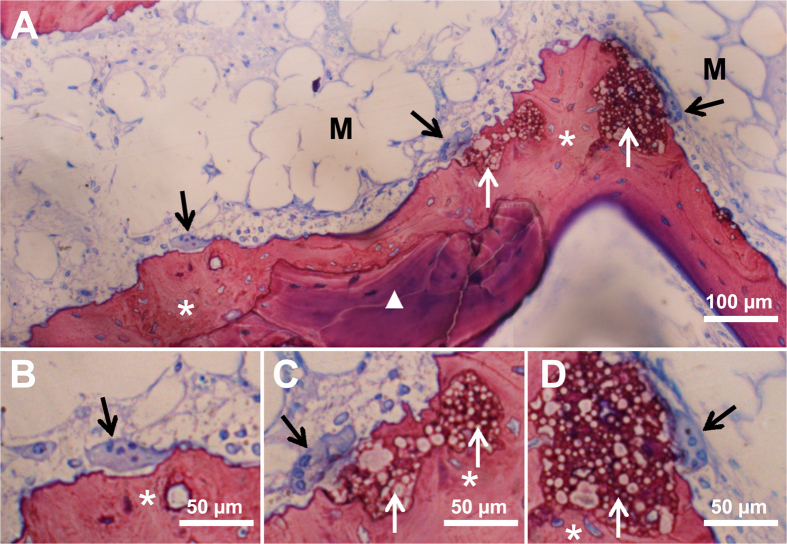
Representative histological images showing new bone formation around BMP2-cop.BioCaP and DBB in DBB + BMP2-cop.BioCaP-group at week 4. (**A**) Bone remodeling process. (**B–D**) High-resolution images of A. Multinucleated cells (black arrow), bone marrow in early stage of formation (M), DBB granules (white triangle), BMP2-cop.BioCaP (white arrow), and newly formed bone (*).

**Figure 8:**
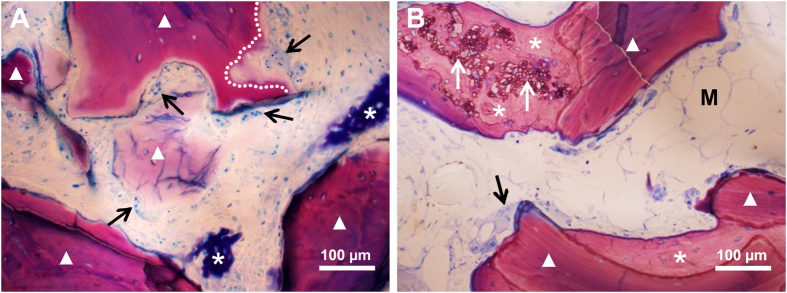
Representative histological images showing multinucleated cells on DBB granules in DBB + BMP2-cop.BioCaP-group at week 4 (**A**), at week 8 (**B**). Multinucleated cells (black arrow), resorption lacunae (white line), newly formed bone (*), BMP2-cop.BioCaP (white arrow), the bone marrow in early stage of formation (M), and DBB (white triangle).

Microscopic images of histological sections showed newly-formed bone, blood vessels in early stage of formation, and bone marrow in early stage of formation close to BMP2-cop.BioCaP in the DBB + BMP2-cop.BioCaP-group at week 4 (Fig. 6A,B). Newly formed bone in DBB + BMP2-cop.BioCaP-group at week 4 in high magnification image is clearly visualized by green pseudo color (Supplementary Fig. 3). New bone in an active remodelling phase with osteoblasts was found in contact with BMP2-cop.BioCaP at 8 weeks (Fig. 6B). Multinucleated cells were observed on the surface of BMP2-cop.BioCaP granules and newly-formed bone at week 4 and 8 (Figs 6A and 7A,C,D). The subsequent bone remodelling might be associated with the presence of these multinucleated cells. Moreover, multinucleated cells were also observed nearby DBB at week 4 (Fig. 8A,B). A light zone on DBB, which indicates demineralization, was often observed beneath the multinucleated cells at week 4 (Fig. 8A). The light zone indicated by a dotted line in Fig. 8A may be a resorption lacuna.

## Discussion

We hypothesized that the novel osteoinducer, BMP2-cop.BioCaP, enhances the therapeutic effect of DBB on the repair of CSBD. We found that BMP2-cop.BioCaP was a biodegradable and efficient osteoinducer. DBB + BMP2-cop.BioCaP robustly enhanced bone formation compared to DBB only, BioCaP only, or DBB + Bio.CaP grafts, respectively. Moreover, DBB + BMP2-cop.BioCaP suppressed the foreign body reaction of DBB. Interestingly, DBB + BMP2-cop.BioCaP grafts were as effective as autologous bone grafts in healing CSBD. These findings indicate the promising therapeutic potential of a combination of BMP2-cop.BioCaP and DBB grafts to repair CSBD.

The possible mechanism of bone defect healing through the use of different bone substitutes is briefly shown in Supplementary Fig. 4. Slow release of BMP2 can enhance osteoinductivity^22^, and shorten the duration of foreign body reaction^23^. In comparison to a burst release, the slow delivery of BMP2 plays a very important role in bone formation^10^,^22^. In a previous study we reported that BMP2 incorporated into BioCaP results in a sustained slow release of BMP2 *in vitro* and its delivery by BMP2-cop.BioCaP leads to a highly efficient osteoinductivity *in vivo*^23^,^24^. In the current study we found the high volume density of osteoblasts on or near DBB as well as high volume of newly formed bone in the DBB + BMP2-cop.BioCaP-group. This confirmed the high efficiency of BMP2-cop.BioCaP using about 10 μg of BMP2, which is 1,000 times less than the amount that is currently applied in the clinic^25^. Sustained slow release of BMP2 in low doses not only increases osteoinduction of the graft material^23^ but also eliminates the adverse effects associated with high doses of BMP2^26^,^27^.

The foreign body reaction and bone remodeling are related to monocytes, macrophages and foreign body giant cells (FBGCs)^28^. Multinucleated cells found in CSBD are osteoclasts and/or FBGCs. Based on our previous study, we assumed that these multinucleated cells could be regarded as an indicator of a foreign body reaction^10^,^23^. We found that BMP2-cop.BioCaP significantly decreased the formation of multinucleated cells on DBB. This finding corroborates the result of our previous study that BMP2-cop.BioCaP suppresses the formation of multinucleated cells in an ectopic environment^23^. In addition, a light zone of DBB appeared beneath the multinucleated cells showing resorption of DBB, which is in accordance with the findings by Mordenfeld and colleagues^18^. It should also be noted that multinucleated cells play a critical role in the degradation of biomaterials^29^. In this study, BMP2-cop.BioCaP-mediated suppression of DBB-induced foreign body reaction has shown a positive correlation with CSBD healing.

All BioCaP particles (with or without BMP2) were observed to be in close contact with the bone or to be entirely encapsulated in the bone. Osteocyte-like mononuclear cells were observed to be in close contact with BMP2-cop.BioCaP. This finding suggests that BMP2-cop.BioCaP is a highly biocompatible material. In a previous study we reported that BMP2-cop.BioCaP particles produced by a biomimetic coating technique are highly osteoinductive and biocompatible in ectopic sites^23^, and bone defects^24^. A number of studies have shown that the coating of Bio.CaP has excellent biocompatibility, biodegradability, and osteoconductivity, respectively, as well as the capability to provide a slow delivery of growth factors such as BMP2 and the vascular endothelial growth factor (VEGF)^10^,^22^,^29^,^30^. CaP microspheres form during the preparation of BMP2-cop.BioCaP^31^,^32^. CaP microspheres serve as the seed for the subsequent growth of a crystalline latticework of octacalcium phosphate under conditions that are conducive to nucleation^33^. Therefore, it is reasonable to speculate that the physico-chemical properties of BMP2-cop.BioCaP might be similar to those of the BioCaP. We found that BioCaP without BMP2 was also conducive to bone formation at 4 weeks after implantation. One possible mechanism for this finding might be related to the degradation of BioCaP, which provides calcium for the process of osteogenesis. The bone retains a direct response to extracellular calcium^34^. Both osteoblasts and osteoclasts use calcium signals as regulators of differentiation and activity.

We observed that 95% of BMP2-cop.BioCaP degraded within 8 weeks. However, BMP2-cop.BioCaP alone did not produce a good repair of the bone defects. The degradation mechanism of BMP2-cop.BioCaP is associated with spontaneous dissolution and cell-based resorption^22^,^23^,^35^. There are two possible mechanisms for the cell-based degradation of material: phagocytosis and acidic degradation^36^. The phagocytosis mechanism is associated with fibroblasts and monocytes/macrophages and the acidic degradation is related to FBGCs and osteoclasts. FBGCs and osteoclasts can produce an acidic microenvironment between the cell membrane and the surface of the biomaterial^27^,^37^. Mononuclear cells, which are osteocyte-like cells, were observed inside the BioCaP particle between the small CaP spheres. This suggests that BioCaP has a porous structure. The porosity also contributes to the degradability of the material^38^. A proper degradability is essential for bone substitutes as scaffolds^39^. Since BMP2-cop.BioCaP degraded too quickly, it could not function as scaffold long enough for the osteoblasts to form new bone. Therefore, BMP2-cop.BioCaP with DBB can be a better bone substitute.

DBB serves as an osteoconductive scaffold for bone formation^12^. In this study, some cell based resorption of DBB was observed, histomorphometric analysis demonstrated its low degradability, which is in accordance with previous findings^13^,^18^. Ideally, bone regeneration requires that the material can be gradually replaced by new bone in a short time^37^. We found that in DBB + BMP2-Cop.BioCaP grafts BMP2-Cop.BioCaP degraded too fast and DBB degraded too slow. This suggests that increasing the ratio of BMP2-Cop.BioCap and DBB in the graft might be more effective treatment modality. Our ongoing study is focused on developing a biodegradable and osteoinductive biomimetic CaP bone graft to treat CSBD in clinic^40^. BMP2-cop.BioCaP alone or combined with DBB enhances new bone formation in ectopic sites in rat^23^. We recently found that BMP2-cop.BioCaP mixed with biphasic calcium phosphate heals cranial bone defect as effective as autologous bone graft in the rat model^24^. In this study, we found that BMP2-cop.BioCaP and DBB healed CSBD in sheep as effectively as autologous bone graft. Due to the limitations related to autografts^4^,^5^,^6^,^7^, we propose that BMP2-cop.BioCaP with a biodegradable graft material may be a potent alternative of autologous bone graft to treat the CSBD in the clinic. However, clinical trials are needed to evaluate this hypothesis.

An ectopic ossification model is useful for testing the principle of an osteoinductive system^33^. However, the osseous environment is different from a non-osseous environment. Our CSBD model in sheep is an excellent animal model for testing biomaterials for use in orthopaedics as well as maxillofacial and dental surgery^23^,^41^. It allows the intraosseous implantation of up to 8 different test materials within one animal due to the standardization of the bone defect, while at the same time it can reduce the overall suffering of animals and yield a necessary sample size to be statistically meaningful^42^,^43^. In addition, results obtained from using sheep are more relevant to humans than those obtained from testing in small laboratory animals because the structure of sheep bones is more similar to the bone structure of humans^44^.

Bone regeneration is a coordinated cascade of events regulated by several growth factors; the local sequential delivery of vascular epithelial growth factor with BMP2 could enhance bone formation compared with only BMP2^45^. Therefore, there is a need to develop a carrier material that will deliver multiple growth factors sequentially and slowly^38^,^46^. Layer-by-layer assembled BioCaP particles appears to be an ideal vehicle for a dual or maybe even a multiple growth factor delivery. By combining different bone growth factors, it will be possible to create a better synthetic alternative to particulate autologous bone. Such a combination will enhance the therapeutic effects of the materials used for filling bone defects during CSBD healing.

## Conclusion

We found that BMP2-cop.BioCaP is a potent osteoinducer as it enhanced bone formation and suppressed the foreign body reaction in DBB grafted CSBD. BMP2-cop.BioCaP also shows good degradability and biocompatibility. These findings suggest that BMP2-cop.BioCaP has promising clinical potential as an osteoinducer to enhance the therapeutic effects of the graft materials for CSBD treatment.

## Materials and Methods

### *In-vitro* preparation of BioCaP particles and BMP2 incorporation

Due to their physico-chemical similarity to bone, BioCaP are widely used as defect filling material during CSBD healing^23^,^24^. In this study, the layer-by-layer assembled BioCaP particles were produced using a well-established biomimetic coating protocol^23^. Briefly, micro-particles of amorphous CaP were formed and deposited by incubating with five-fold concentrated simulated body fluid (684 mM NaCl; 12.5 mM CaCl_2_; 21 mM NaHCO_3_; 5 mM Na_2_HPO_4_ and 7.5 mM MgCl_2_) for 24 hours at 37 °C. These particles (size 5–20 μM) served as the cores for the subsequent layer-by-layer assembly. They were immersed in 1000 ml of a supersaturated CaP solution [40 mM HCl; 2 mM Na_2_HPO_4_; 4 mM CaCl_2_; 50 mM TRIS-HCl (pH 7.4)] for 48 hours at 37 °C. The first layer of crystalline CaP coating deposited on the amorphous CaP micro-particles. Then these particles were immersed in the five-fold concentrated simulated body fluid for 24 hours and subsequently in supersaturated CaP solution for 48 hours for second layer of coating. Consequently, the size of BioCaP particles was enlarged by assembling layer-by-layer. Molar ratio of CaP crystalline particles prepared by this procedure ranges between 1.34–1.67^47^.

BioCaP particles were assembled in three cycles. During the preparation of the outer layer, BMP2 (INFUSE® Bone Graft, Medtronic, USA) was added to the supersaturated CaP solution to a final concentration of 2 μg/ml and co-precipitated into the outermost crystalline CaP layer (BMP2-cop.BioCaP). The BMP2-cop.BioCaP particles were then freeze dried and retrieved with a size of 0.25 to 1 mm. The entire procedure was conducted under sterile conditions.

### Quantification of the amount of the incorporated BMP2

The amount of BMP2 incorporated to Bio.CaP was determined by a commercially available enzyme-linked immunosorbent assay (ELISA) kit (PeproTech, London, UK) as described previously^23^,^24^,^35^. Briefly, 0.07 g of BMP2-cop.BioCaP (n = 6) was dissolved in 1 ml 0.5 M EDTA (pH 8.0). The ELISA assay was performed according to the manufacturer’s instructions.

### Surface characterization of BioCaP

The surface characteristic of BioCaP with or without incorporated BMP2 was monitored using a scanning electron microscope (SEM, XL 30, Philips, The Netherlands). For this purpose, samples of the material were mounted on aluminum stubs and sputtered with gold particles to a thickness of 10–15 nm.

### Experimental groups

Eight groups of implantation sites with different treatments were studied with six sheep in each group (Supplementary Table 1).

1. No treatment in the empty defect (empty defect-group: negative control).
2. Autologous bone particles (autologous bone graft-group: positive control).
3. DBB granules alone (DBB-group: negative control for BMP2-cop.BioCaP-group).
4. DBB granules with surface-adsorbed BMP2 (DBB + BMP2-group: control for BMP2-cop.BioCaP-group).
5. BioCaP particles (BioCaP-group: negative control for the effects of BMP2).
6. BMP2-cop.BioCaP particles (BMP2-cop.BioCaP-group: experimental group).
7. DBB granules mixed with BioCaP particles (DBB + BioCaP-group: negative control for DBB + BMP2-cop.BioCaP-group).
8. DBB granules mixed with BMP2-cop.BioCaP particles (DBB + BMP2-cop.BioCaP-group: experimental group).

Autologous bone was harvested from the cylindrical bone defects during the surgery and ground to 0.25–1 mm particles. The amount of BMP2 (10.0 μg/graft) used was determined according to previous studies^22^,^23^,^24^,^27^,^48^.

### Experimental animal model

A total of 12 adult female Australian sheep, which were 2 to 4 years old and weighed between 40 to 50 kg, were used in the present study, which was approved by Ethical Committee of School of Stomatology in Zhejiang University. All the animal experiments were carried out according to the ethics laws and regulations of P.R. China. Throughout the study, the sheep were treated following the guidelines of animal care established by Zhejiang University.

The sheep were anaesthetized by administering Sumianxin II (0.3 ml/kg), penicillin (5 × 10^4^ U/kg), and atropine (0.03 mg/kg). A local anaesthesia (1% lidocaine with 1:100,000 adrenaline) and skin disinfection (0.5% iodophor solution) were applied to the implantation sites. The implantation sites were the proximal part of the diaphysis and distal epiphysis of humerus and femur of sheep^40^. The implantation sites were assigned to the eight groups with 6 sheep in each group following a randomization protocol^49^.

Cylindrical defects 8 mm in diameter and 13 mm deep were created as described in a previous study^40^. Membranes (Bio-Gide^®^, Geistlich, Switzerland) were used to cover the defects. Samples with surrounding tissues were taken by surgery after 4 and 8 weeks of implantation. All the sheep exhibited good health and all the surgical implant sites had healed well without any complications. There were no visual signs of inflammation or adverse tissue reactions.

### Histological procedures

Samples with surrounding tissue were fixed chemically and embedded into block as reported previously^10^,^33^. Applying a systematic random sampling strategy^50^, the samples were sawn vertically to the long axis into 10 slices of 600 μm thickness at an interval of 1 mm. All the slices from each sample were mounted separately on plexiglass holders and polished. The slices were surface stained with McNeal’s Tetrachrome, basic fuchsine and toluidine blue^10^, and examined with a light microscope (Leica). McNeal’s Tetrachrome, basic fuchsine and toluidine blue stained old bone with reddish and newly formed bone with purple color. There was not strong color contrast between the newly formed bone, old bone or graft material. Therefore to better clarify the global changes of newly formed bone form old bone in different treatment groups, we painted the histological images by pseudo color (green).

### Histomorphometric analysis

The space under the fibrous capsule that embraced the whole block of implants (subcapsular space) was taken as the reference space. This involves measuring the cross-sectional area of a defined number of tissue sections at a fixed distance apart through the reference volume^10^,^23^,^24^. Ten slices of each sample were used for a quantitative histomorphometric analysis. The volume of the newly formed bone, BioCaP, and DBB were measured stereologically from their area density on tissue sections using the point-counting technique^23^,^51^. The volume density of multinucleated cells and osteoblasts on or near the DBB were also analyzed.

The volume density of multinucleated cells and osteoblasts on or near DBB was normalized to the volume of DBB. The volume density of multinucleated cells or osteoblasts (D) is defined as its volume (V_a_) per unit volume of DBB (V_b_): D = V_a_/V_b_. The degradation of the DDB and BioCaP was evaluated by measuring their volume before implantation (V_time0_) and 4 and 8 weeks later (V_4/8 weeks_) using the same histological method. Six samples of 0.07 cm^3^ containing BioCaP were reserved for ‘time 0’. The percentage (P) of material that is not degraded was then calculated as P = V_4/8weeks_/V_time0_ × 100%.

### Statistical analysis

All data are presented as mean values with the standard deviation (mean ± SD). Data were compared using a one way analysis of variance (ANOVA), and post hoc comparisons were made using Bonferroni’s corrections. Each *p* value was adjusted to account for multiple comparisons. The significance level was set at *p* < 0.05.

## Additional Information

**How to cite this article**: Liu, T. *et al*. BMP2-coprecipitated calcium phosphate granules enhance osteoinductivity of deproteinized bovine bone, and bone formation during critical-sized bone defect healing. *Sci. Rep.* **7**, 41800; doi: 10.1038/srep41800 (2017).

**Publisher's note:** Springer Nature remains neutral with regard to jurisdictional claims in published maps and institutional affiliations.

## Supplementary Material

Supplementary Dataset 1
