# Supplementary material for: BMP2-coprecipitated calcium phosphate granules enhance osteoinductivity of deproteinized bovine bone, and bone formation during critical-sized bone defect healing
Source: Sci Rep. 2017 Jan 31;7:41800. doi: 10.1038/srep41800 (PMC5282552; doi:10.1038/srep41800)
Supplement: Supplementary Dataset 1 [file srep41800-s1.doc]

**Supplementary data**

Atricle title: BMP2-coprecipitated calcium phosphate granules enhance osteoinductivity of deproteinized bovine bone, and bone formation during critical-sized bone defect healing

Journal name: Scientific Reports

Author names: Tie Liu, Yuanna Zheng, Gang Wu, Daniel Wismeijer, Janak L. Pathak, Yuelian Liu,

**Supplementary figure 1**


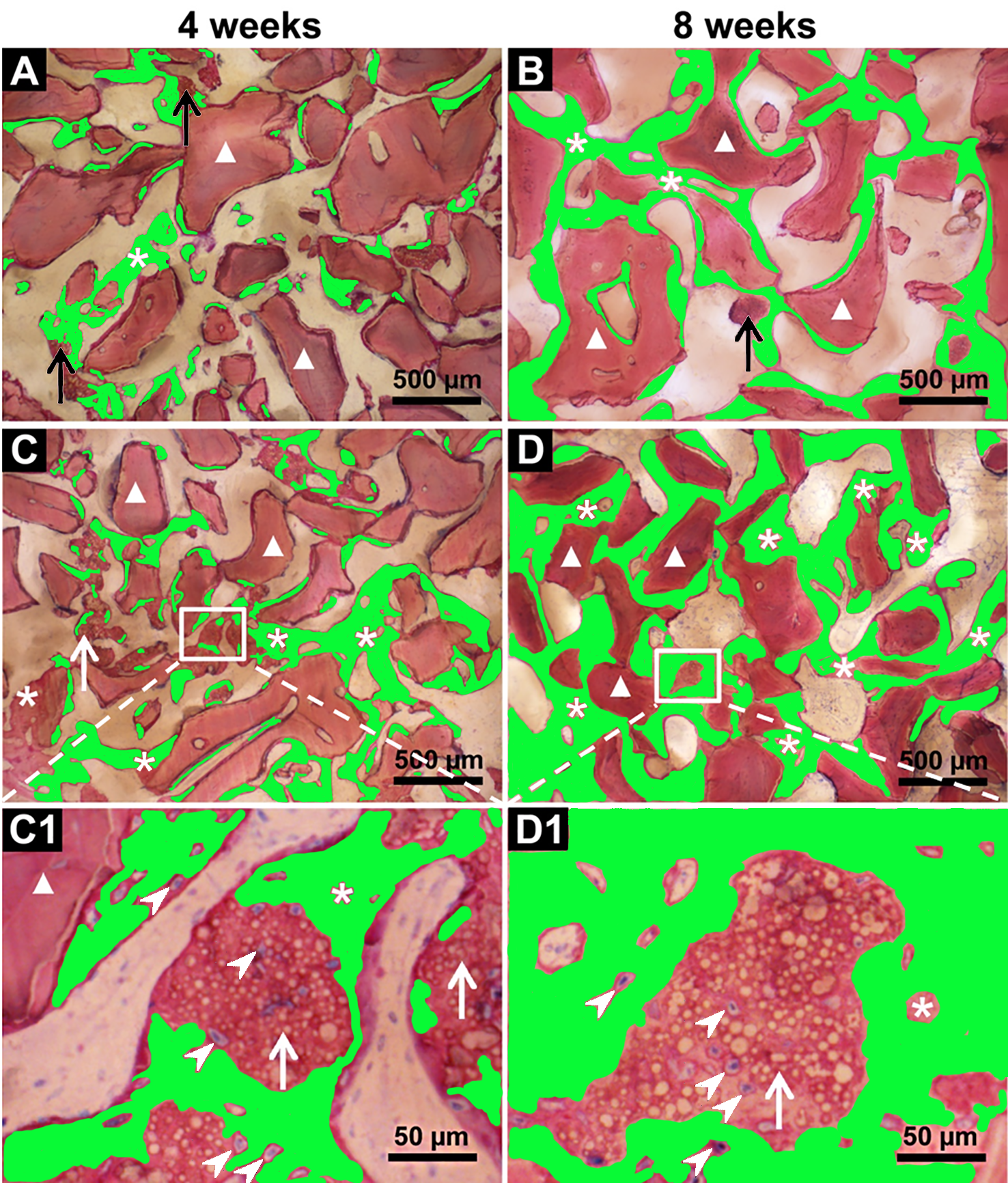


**Supplementary Figure 1. Newly formed bone painted by pseudocolor (green)** **in Fig. 4** (A) DBB+BioCaP-group at week 4. (B) DBB+BioCaP-group at week 8. (C) DBB+BMP2-cop.BioCaP-group at week 4. (D) DBB+BMP2-cop.BioCaP-group at week 8. (C1) high-resolution image of C. (D1) high-resolution image of D. BioCap (black arrow), BMP2-cop.BioCaP (white arrow), BioCaP or BMP2-cop.BioCaP in close contact with newly formed bone (*), osteocyte-like cells (white arrow heads), and DBB in close contact with newly formed bone (white triangle).

**Supplementary figure 2**


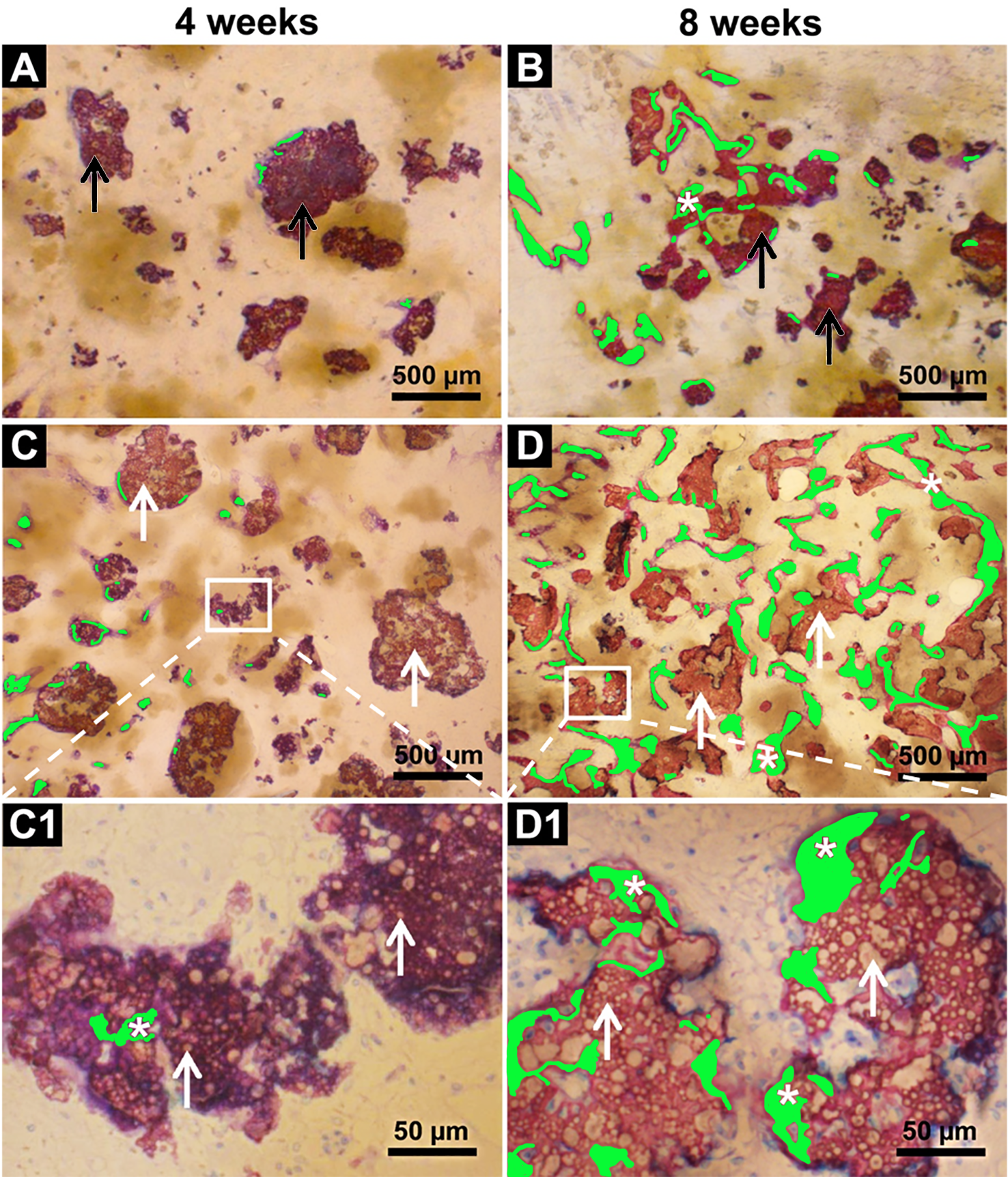


**Supplementary figure 2. Newly formed bone painted by pseudocolor (green)** **in Fig. 5.** (A) BioCaP-group at week 4. (B) BioCaP-group at week 8. (C) BMP2-cop.BioCaP-group at week 4. (D) BMP2-cop.BioCaP-group at week 8. (C1) High-resolution image of C. (D1) High-resolution image of D. BioCaP (black arrow), BMP2-cop.BioCaP (white arrow), BioCaP or BMP2-cop.BioCaP in close contact with newly formed bone (*), unmineralized newly formed bone (purple color), and mineralized and mature newly formed bone (reddish color).

**Supplementary figure 3**


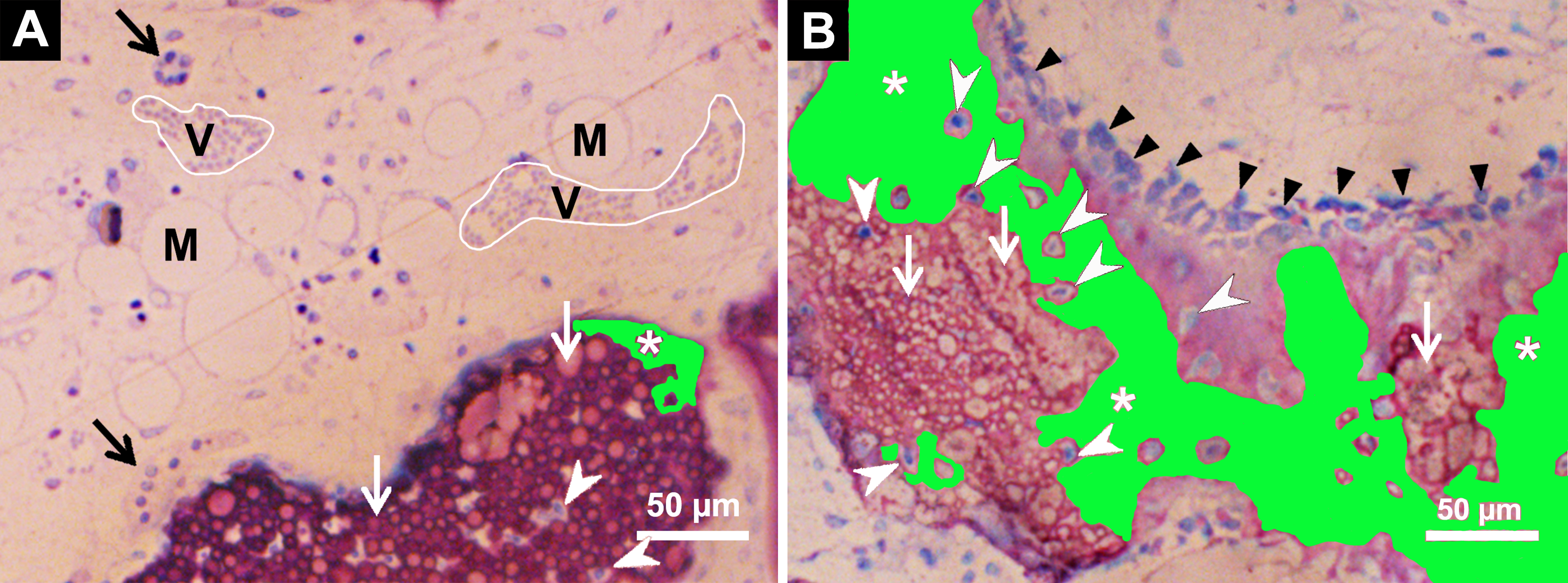


**Supplementary figure 3. Newly formed bone painted by pseudocolor (green)** **in high magnification image of DBB+BMP2-cop.BioCaP group at week 4.** (A) The blood vessels in early stage of formation (V) and the bone marrow in early stage of formation (M) close to BMP2-cop.BioCaP. (B) An active phase of bone formation with osteoblasts (black triangles), osteocyte-like cells (white arrowheads) in close contact with or in the interior of the BMP2-cop.BioCaP particles. The slices were surface-stained with McNeal's Tetrachrome, basic fuchsine and toluidine blue. BMP2-cop.BioCaP (white arrow), and multinucleated cells (black arrow).

**Supplementary figure 4**


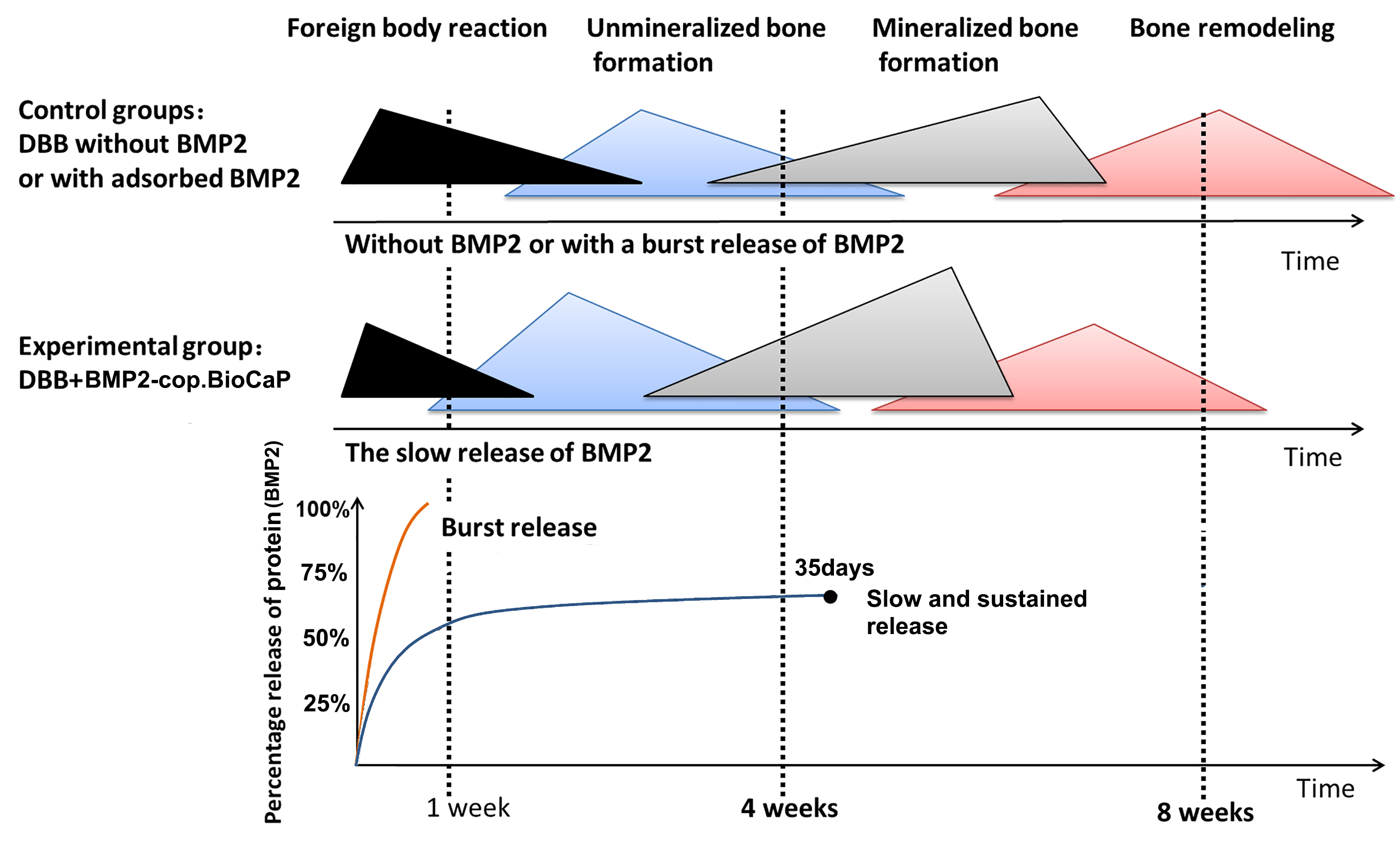


**Supplementary figure 4.** Schematic graphs describing the different stages of the bone defect repair by using different bone substitutes and the possible effect of BMP2 in this process. The relative periods of time for each of the phases of bone recovery are represented by triangles, demonstrating their intensity during the repair. The possible release kinetics of BMP2 was described including a burst release and a slow release.

**Supplementary Table 1**

**Supplementary Table 1:Volume of graft material per sample and dose of BMP2 used in this study**

| **Groups** | | **Graft materials** | **Total Loading** |
| --- | --- | --- | --- |
|  |  | **Volume of graft material per sample** | **Dose of BMP2 (per sample)** |
| **(1)** | **No treatment** | **-** | **-** |
| **(2)** | **Autologous bone** | **0.66cm^3^** | **-** |
| **(3)** | **DBB** | **0.66cm^3^** | **-** |
| **(4)** | **DBB+BMP2** | **0.66cm^3^** | **35μg** |
| **(5)** | **BioCaP** | **0.66cm^3^** |  |
| **(6)** | **BMP2-cop.BioCaP** | **0.66 cm^3^;** | **10.3μg** |
| **(7)** | **DBB+BioCaP** | **BioCaP 0.07 cm^3^;**  **DBB 0.59 cm^3^** | **-** |
| **(8)** | **DBB+BMP2-cop.BioCaP** | **BMP2-cop.BioCaP 0.07cm^3^;**  **DBB 0.59cm^3^** | **10.3μg** |

DBB, deproteinised bovine bone; BMP, bone morphogenic protein; BioCaP, layer-by-layer assembled biomimetic calcium phosphate granules.

**Supplementary Table 2**

Supplementary Table 2: ELISA results showing incorporation of BMP2 in Bio.CaP particles

| **BMP2-Cop.BioCaP** | **Concentration of**  **BMP2 (μg)/mg**  **of Bio.CaP** | **Concentration of BMP2 (μg)/70mg**  **of Bio.CaP** |
| --- | --- | --- |
| **Sample 1** | **0.177** | **12.39** |
| **Sample 2** | **0.136** | **9.52** |
| **Sample 3** | **0.125** | **8.75** |
| **Sample 4** | **0.148** | **10.36** |
| **Sample 5** | **0.117** | **8.19** |
| **Sample 6** | **0.181** | **12.67** |
|  |  | **Mean±SD, 10.3±1.9** |
